# Reversible Gynecomastia Induced by Darbepoetin in a Patient With Chronic Kidney Disease

**DOI:** 10.1155/crin/6653329

**Published:** 2025-04-06

**Authors:** Randah Abdullah Dahlan, Maram Khalid Sait, Alaa Khalil Monjed

**Affiliations:** ^1^Department of Internal Medicine, King Abdullah Medical City, Mecca, Makkah Province, Saudi Arabia; ^2^Department of Internal Medicine, King's College Hospital London, Jeddah, Makkah Province, Saudi Arabia

**Keywords:** chronic kidney disease, darbepoetin, erythrocytes-stimulating agents, gynecomastia

## Abstract

Gynecomastia is a benign proliferation of the glandular tissue of the male breast. It is a common physiological condition during infancy and puberty and typically resolves spontaneously in most cases. However, adult-onset gynecomastia requires proper evaluation to identify the underlying cause. Several medications are associated with gynecomastia in adults, with some having a clear pathophysiologic mechanism while others do not. We report a 78-year-old male with chronic kidney disease (CKD) who developed painful gynecomastia following the administration of darbepoetin. The gynecomastia resolved upon cessation of darbepoetin but recurred upon its reintroduction. Extensive evaluation did not identify any other underlying cause. To our knowledge, this is the first case describing an association between darbepoetin and gynecomastia. In addition to presenting the case, we briefly discuss the hormonal changes in CKD patients and the potential effects of synthetic erythropoiesis-stimulating agents (ESAs) on hormone regulation.

## 1. Introduction

Proper evaluation of gynecomastia developing in adults and elderly men may reveal an underlying pathology in approximately 45%–50% of the cases [1]. There are several causes of gynecomastia, but regardless of the underlying cause, the common pathophysiology is an increase in the ratio of estrogen to androgen balance [1]. Drug-induced gynecomastia accounts for up to 25% of the cases [2, 3]. Some of these drugs cause gynecomastia by affecting the numerator of the estrogen-to-androgen ratio (i.e., enhancing the production of estrogen or having intrinsic estrogenic properties), while other drugs affect the denominator of the ratio (i.e., hindering the synthesis, action, or metabolism of androgens) [1]. However, the mechanism by which some well-known gynecomastia-associated drugs (e.g., spironolactone) affect the estrogen-to-androgen ratio is not clear [1]. Furthermore, it is sometimes not clear whether it is the drug or the underlying disease itself that causes gynecomastia [1]. Therefore, suspecting a certain drug as a potential cause should not preclude a detailed investigation.

We describe a patient with CKD who developed new-onset painful gynecomastia after the initiation of darbepoetin. His symptoms improved after stopping darbepoetin, but symptoms worsened again after reintroducing darbepoetin. The association between darbepoetin and gynecomastia has not been described before. In addition to describing the case and the performed workup, we outline the hormonal effects of ESAs in patients with CKD.

## 2. Case Presentation

A 78-year-old male attended the nephrology clinic in June 2023 for follow-up of his CKD. Besides CKD, he also had long-standing diabetes mellitus, hypertension, dyslipidemia, and ischemic heart disease. His medication list included hydralazine, isosorbide dinitrate, bisoprolol, aspirin, clopidogrel, pantoprazole, vildagliptin, furosemide, atorvastatin, sodium bicarbonate, and calcium carbonate tablets. During the clinic visit, the management of his severe anemia (hemoglobin level of 7.3 mmol/L) was discussed, and he was initiated on subcutaneous darbepoetin injections at 40 mcg per week after mentioning that he had recently received intravenous iron in a private polyclinic. He was also referred to gastroenterology for consideration of upper and lower endoscopy as part of the workup of his severe anemia.

He returned 6 weeks later for follow-up, and his main complaint was bilateral painful breast swelling that started 2 weeks after the last visit. He insisted that his complaint was attributed to the darbepoetin injections, and he admitted skipping the last due dose. He stated that he had not started any new medication and had not changed the dose of any of his regular medications. His physical examination revealed bilaterally prominent, enlarged breasts, more on the right side, with no overlying skin changes. Palpating the breasts revealed bilaterally tender, rubbery, glandular, mobile tissue beneath the areolar area. His investigations at that time are shown in Table 1.A.

As his hemoglobin improved to 10.3 (mmol/L) from 7.3 (mmol/L), darbepoetin was not prescribed, and he was advised to see his family physician for further workup of his breast swelling. At his next follow-up visit in October 2023, he had no active complaint and reported that he declined the upper and lower endoscopy procedures offered by the gastroenterology team, and he did not see his family physician for his breast swelling as his symptoms had improved and almost resolved. His hemoglobin had dropped again to 7.4 (mmol/L) with adequate iron stores, so he was advised to consider undergoing the upper and lower scope and to resume darbepoetin.

However, when he returned for follow-up after 2 months, he reported developing painful bilateral breast swelling again after taking two doses of darbepoetin, but this time, it was more pronounced on the left side. He stopped taking it and felt better after that. The plan at that time included ordering an ultrasound of his breasts, referring him to endocrinology, and advising him to resume darbepoetin for the time being because of his medical need and the lack of any known association between his symptoms and darbepoetin. Two months later, he reported that his painful breast swelling had recurred when he resumed darbepoetin, so he stopped it a month after his last visit. He felt better after stopping it and refused to take it again in the future. The results of his hormonal workup and the breast ultrasound are listed in Tables 1.B and C. During the follow-up visits, the option of trying epoetin alfa was discussed; however, as it was not available at our institution, the patient arranged to bring it from another hospital. He tolerated it very well with no complaints during the following 6 months.

## 3. Discussion

Patients with CKD may develop gynecomastia due to CKD-associated hormonal dysfunction, including secondary hypogonadism resulting from suppression of testosterone production, elevated follicle-stimulating hormone (FSH) and luteinizing hormone (LH), and/or hyperprolactinemia caused by decreased renal clearance [1, 4, 5]. Moreover, CKD patients frequently use drugs that may themselves cause gynecomastia (e.g., calcium channel blockers, proton pump inhibitors, angiotensin-converting enzyme inhibitors, and spironolactone) [1, 4]. The utilization of erythrocyte-stimulating agents (ESAs) has been reported to have some positive effects on the hormonal profile of renal patients. For example, some data suggest that erythropoietin therapy in chronic hemodialysis patients is associated with normalization of prolactin levels [6]. However, it is unclear whether this normalization results from the correction of anemia or from a specific effect of erythropoietin itself [6]. Some other data indicate that sexual function may improve in male chronic hemodialysis patients during erythropoietin therapy without a significant effect on the levels of sexual hormones (testosterone or estradiol), prolactin, LH, and FSH hormones [7]. Other data show that if any hormonal changes occur with erythropoietin therapy in patients with CKD, these include an increase in the plasma levels of testosterone, a decrease in levels of FSH and LH, and normalization of prolactin and FSH levels [8]. Despite these findings, the repeated occurrence of symptoms each time our described patient used darbepoetin, with repeated improvement observed after its cessation, suggests a potential association that must be taken into consideration. This is particularly true in the absence of another explanation, with stable renal function during that period and no other new medications added or dosage changes. The potential additive effect of coadministered medications on the development of gynecomastia has been reported in some cases [9, 10]. While this additive effect cannot be ruled out in the current case, it also cannot be confirmed based on the available data. The patient's hormonal evaluation clearly indicated no underlying hormonal cause for his gynecomastia, such as hyperthyroidism, hyperprolactinemia, or hypogonadism. The rise in LH and SHBG levels is likely attributable to the natural aging process. The fact that the patient did not develop the same symptoms when using epoetin alfa suggests that it is a drug-specific effect rather than a class effect.

No pathophysiological explanation can be provided for the development of gynecomastia when using darbepoetin. However, this lack of explanation is also seen with some other drugs known to cause gynecomastia (e.g., spironolactone) [1].

In summary, this case raises the possibility of a potential association between the development of gynecomastia and the use of darbepoetin. To our knowledge, this association has never been described before. Clinicians should remain aware of this possibility, and reporting similar observations is encouraged.

## Figures and Tables

**Table 1:** Index case investigations.

Test item	Reference range	Result
*A: Patients' initial investigations*		
Hemoglobin (mmol/L)	13.5–17.5	10.3
Platelet (× 10^9^/L)	150–400	381
White blood cells (× 10^9^/L)	3.9–11	6.2
Ferritin (pmol/L)	48.9–617	665.1
Transferrin saturation (%)	5%–14%	40%
Sodium (mmol/L)	136–145	139
Potassium (mmol/L)	3.5–5.1	4.2
Bicarbonate (mmol/L)	22–29	23.9
Blood urea nitrogen (mmol/L)	2.86–8.57	10.71
Creatinine (mmol/L)	65.4–119.34	399.6
Corrected calcium (mmol/L)	2.12–2.52	2.02
Phosphorus (mmol/L)	0.81–1.45	1.65
Albumin (g/L)	35–5.2	47
Total bilirubin (μmol/L)	< 20.5	10.26
Alkaline phosphatase (Unit/L)	40.0–150.0	97
Aspartate aminotransferase (Unit/L)	8.0–48	10.0
Alanine aminotransferase (Unit/L)	16–63	16
Parathyroid hormone (ng/L)	15–68.3	233.3

*B: Patients' hormonal studies*		
Testosterone, free (pmol/L)	8.6–61.7	47.1
Testosterone, total (nmol/L)	8.35–28.76	22.7
Estradiol (pmol/L)	0.0–146.1	99.8
Sex hormone binding globulin (nmol/L)	20.0–77.0	101
Luteinizing hormone (IU/L)	1.5–9.3	20.56
Follicle stimulating hormone (IU/L)	1.4–18.1	8.14
Thyroid stimulating hormone (mIU/L)	0.35–4.94	2.90
T4 free thyroxine (pmol/L)	11.4–22.6	12.3
Human chorionic gonadotropin quantitative (IU/L)	< 5	< 2.3
Prolactin (μg/L)	3.46–19.4	13.9

*C: Ultrasound of the breasts*		
Ultrasound of breasts: centralized well-defined retro areolar hypoechoic flame shape appearance glandular tissue in keeping with gynecomastia, with breast imaging-reporting and data system (BI-RADS) assessment category 2		

## Data Availability

The data that support the findings of this study are available from the corresponding author upon reasonable request.

## References

[1] Kanakis G. A., Nordkap L., Bang A. K. (2019). EAA Clinical Practice Guidelines-Gynecomastia Evaluation and Management. *Andrology*.

[2] Eckman A., Dobs A. (2008). Drug-induced Gynecomastia. *Expert Opinion on Drug Safety*.

[3] Batteux B., Llopis B., Muller C. (2020). The Drugs that Mostly Frequently Induce Gynecomastia: A National Case – Noncase Study. *Therapie*.

[4] Dickson G. (2012). Gynecomastia. *American Family Physician*.

[5] Holdsworth S., Atkins R. C., de Kretser D. M. (1977). The Pituitary-Testicular axis in Men with Chronic Renal Failure. *New England Journal of Medicine*.

[6] Schaefer R. M., Kokot F., Kuerner B., Zech M., Heidland A. (1989). Normalization of Serum Prolactin Levels in Hemodialysis Patients on Recombinant Human Erythropoietin. *The International Journal of Artificial Organs*.

[7] Bommer J., Kugel M., Schwöbel B., Ritz E., Barth H. P., Seelig R. (1990). Improved Sexual Function during Recombinant Human Erythropoietin Therapy. *Nephrology Dialysis Transplantation*.

[8] Iglesias P., Carrero J. J., Dıez J. J. (2012). Gonadal Dysfunction in Men with Chronic Kidney Disease: Clinical Features, Prognostic Implications and Therapeutic Options. *Journal of Nephrology*.

[9] Kaufman K. R., Podolsky D., Greenman D., Madraswala R. (2013). Antidepressant-selective Gynecomastia. *The Annals of Pharmacotherapy*.

[10] Rahman S., Aiman U., Haseeen M. (2009). Gynecomastia: An ADR Due to Drug Interaction. *Indian Journal of Pharmacology*.

